# Where is the PrEP for migrants?: Displaced Venezuelans, and other refugee populations are at risk of HIV infection. Providing PrEP and tackling stigma are critical to prevent this, writes Rebecca Irons

**DOI:** 10.1136/bmj.q315

**Published:** 2024-02-07

**Authors:** Rebecca Irons

**Affiliations:** University College London

With more than 7 million displaced people, the Venezuelan refugee crisis, fuelled by political, economic, and social instability, is on a similar scale to that of Ukraine and Syria yet remains out of international headlines^1^. Despite the Venezuelan health system reaching near collapse^2^, medical and humanitarian aid have steadily declined over recent years, with an acceleration in 2021 as international agencies redirected funds towards Ukraine^3^.

To prevent the spread of HIV, access to sexual and reproductive health services for displaced people is essential. An estimated 8,000 Venezuelan migrants are living with HIV^4^, mostly in neighbouring Latin American countries. To stem the spread of HIV and attend to migrant health, PrEP (pre-exposure prophylaxis) must urgently be made available to Venezuelans in Latin America, and to other migrant populations across the globe. These populations may be facing a similar stigma as a barrier to healthcare. They continue to rely on the support of non-governmental organisations and aid agencies who provide anti-retroviral therapy until they can access state-funded healthcare. But PrEP continues to be unavailable to these migrants.

The lack of PrEP availability to migrants may be closely related to stigmatised perceptions of who is transmitting HIV. A myriad of factors mean that Venezuelans are often stigmatised as vectors for sexually transmitted diseases and as a risk to local populations in Latin America^5^. With the country’s shortage of anti-retroviral therapy between 2017-2018, there are perceptions that the virus has run rampant in Venezuela^6^. This could lead to the misconception that it is host populations that are at risk from Venezuelans, not the other way around. This view and associated stigma[?] may result in cases of vulnerable migrants struggling or being unable to negotiate use of contraception in serodiscordant relationships and, in the absence of PrEP, being infected with HIV themselves.

In Latin America demand for PrEP has increased over recent years^7^, though only 11 of 17 countries in the region offer it as of 2022^8^, Venezuela’s neighbour and the nation with the highest intake of migrants, Colombia, is one of them^9^. Since being approved in 2019, studies suggest that some reticence towards prescribing PrEP remains among Colombian physicians^10^, with perceived risk of HIV exposure being a key factor in willingness to prescribe. From a policy perspective, Venezuelan migrants are not recognised to be at high risk of HIV infection and at present no international aid agency or local healthcare provider offers PrEP to this population. This is an error, and migrants must be considered as a key population at risk of HIV infection and in need of access to PrEP. Health-related data from Venezuela, and about Venezuelan migrants, is difficult to obtain. But evidence of lived experience and social contributory factors highlights this need.

In my research I came across a case of women who migrated from Venezuela andhad been in Colombia for a few years when she met her Colombian boyfriend, who was HIV positive. Though effort was made to seek PrEP for her, this was unavailable due to her “refugee” status. The couple relied on condom-use and she also ended up contracting HIV. In cases such as this where the status of the partner is known and when anti-retroviral therapy is unavailable or not adhered to so as to reach undetectability, PrEP should be offered to the partner to avoid infection. Clearly Venezuelans need PrEP as much as Colombians, so why is it not available?

The same issue is relevant to other migrant populations. For example, Poland presently hosts the largest number of Ukrainian refugees^11^ but it does not reimburse PrEP and lacks formal implementation for the population^12^. This suggests that Ukrainians who engage in unprotected sexual intercourse are at risk of HIV infection with limited access to PrEP. Indeed, HIV is reportedly spreading in Ukraine due to the war’s impact on healthcare services^13^ and refugee women are being sexually propositioned by host-country men^14^.

To tackle these intersecting issues, I recommend that healthcare providers in migrant host countries urgently seek to secure adequate provision of PrEP for all those that may be at risk of HIV exposure. For this to be effective it will be necessary to tackle deep-seated stigmatisation of migrants as “HIV vectors” and instead adopt a nuanced understanding of host-migrant relations and risk factors in sexual health.

## Footnotes

1 UNHCR (2023). Venezuela Situation, https://www.unhcr.org/emergencies/venezuela-situation

2 Shannon Doocy, Kathleen R Page, Charissa Liu, Hayley Hoaglund, and Daniela C Rodríguez. (2022). Venezuela: out of the headlines but still in crisis. *Bull World Health Organ*. 100(8): 466–466A

3 Bryan Pietsch and Hari Raj. (2022). Venezuela’s refugee crisis similar to Ukraine’s in scale, but not aid. *The Washington Post*, September 4, 2022 https://www.washingtonpost.com/world/2022/09/04/venezuela-refugee-crisis-ukraine-syria/

4 Patrick Ammerman. (2019). Venezuela’s HIV Crisis Crosses the Border. *Foreign Policy* August 28, 2019 https://foreignpolicy.com/2019/08/28/venezuela-hiv-crisis-crosses-the-border-colombia-migrants-health-care/

5 Rebecca Irons (2022) ‘Aquí viene una Veneca más’: Venezuelan migrants and ‘the sexual question’ in Peru, *Anthropology & Medicine*, 29:3, 323-337, DOI: 10.1080/13648470.2022.2046700

6 Dimitri O’Donnell. 2017. ‘No Hope Left in Venezuela’ for Those With HIV, Advocates Say. *NBC News* Aug. 25, 2017 https://www.nbcnews.com/feature/nbc-out/no-hope-left-venezuela-those-hiv-advocates-say-n795866

7 Lorena Guerrero-Torres and Juan G Sierra-Madero. (2022). Implementation of PrEP in Latin America. *The Lancet HIV* December 21 2022

8 Jerome T Galea, Ricardo Baruch and Brandon Brown. (2018). ¡PrEP Ya! Latin America wants PrEP, and Brazil leads the way. *The Lancet HIV* 18 February 2018

9 Jorge Martínez-Cajas, Beatriz Alvarado-Llano, Ernesto Martínez-Buitrago, Julian Torres-Isasiga, Marcela Arrivillaga, Pilar Camargo, Ximena Galindo-Orrego, Héctor Mueses-Marín. (2022). AC-2020-12-1420.R1 – HIV care providers’ familiarity, concerns, and attitudes about HIV PrEP in Colombia: insights from the PrEP-Col-Study. *AIDS Care* 2022 Nov;34(11):1428-1434. doi: 10.1080/09540121.2022.2029813

10 Jorge Luis Martinez-Cajas, Julian Torres, Hector Fabio Mueses, Pilar Camargo Plazas, Marcela Arrivillaga, Sheila Andrea Gomez, Ximena Galindo, Ernesto Martinez Buitrago & Beatriz Eugenia Alvarado Llano. (2022). Applying implementation science frameworks to identify factors that influence the intention of healthcare providers to offer PrEP care and advocate for PrEP in HIV clinics in Colombia: a cross-sectional study. *Implementation Science Communications* volume 3, Article number: 31 2022

11 David Blood (2022) UK takes in fewer Ukrainians per capita than most of Europe, *The Guardian* Friday 3^rd^ June 2022

12 Roger Pebody (2019) Some European governments are paying €3 a month for PrEP, while others are paying €850, *AIDSMAP* 5 November 2019

13 The Global Fund (2022) Thematic Report: War in Ukraine: Maintaining Lifesaving HIV and TB Services, *The Global Fund*

14 Rajeev Syal (2022) Stop matching lone female Ukraine refugees with single men, UK told, *The Guardian* 13 Apr 2022 https://www.theguardian.com/world/2022/apr/13/stop-matching-lone-female-ukraine-refugees-with-single-men-uk-told

